# Motivations for Using Dietary Supplements in Elite Ice Hockey—Controlling Weight and Enhancing Performance

**DOI:** 10.3390/nu16162667

**Published:** 2024-08-13

**Authors:** Sofie Christensen, Astrid Gjelstad, Ingunn Björnsdottir, Fredrik Lauritzen

**Affiliations:** 1 Science and Medicine, Anti-Doping Norway, 0855 Oslo, Norway; 2 Section for Pharmaceutics and Social Pharmacy, Department of Pharmacy, University of Oslo, 0316 Oslo, Norway; ingunn.bjornsdottir@farmasi.uio.no; 3 Section for Pharmaceutical Chemistry, Department of Pharmacy, University of Oslo, 0316 Oslo, Norway

**Keywords:** anti-doping, ice hockey, dietary supplements, unintentional doping, nutrition, performance, weight regulation

## Abstract

*Background:* Excessive or improper use of dietary supplements (DSs) by athletes may cause adverse effects, such as impaired performance or failing a doping test, making it important for athletes to mitigate risk and make well-informed choices when using supplements. *Methods:* This study used focus group interviews to examine the attitudes, motivations, and practices related to DSs among male elite ice hockey players. *Results:* The players used a wide range of products, ranging from vitamins to multi-ingredient pre-workout supplements. Consuming DSs was considered as a practical and convenient way to ingest sufficient calories to gain or maintain the body weight and muscle mass needed to meet the physical requirements of the sport. The athletes demonstrated a lenient and ignorant attitude when acquiring and using supplements, with a non-critical trust in the guidance provided to them by the coach or physician. Having completed basic anti-doping education in the form of an e-learning program did not appear to result in taking a more careful approach to using DSs. *Conclusions:* Through their DS practices, elite ice hockey players may put themselves at risk for anti-doping rule violations. A comprehensive approach is needed when aiming to prevent unintentional doping in this athlete cohort.

## 1. Introduction

Adequate and personalized nutrition is essential for optimal sport performance [1]. While a “food first” approach is recommended by most experts, dietary supplements (DSs) can be beneficial in certain situations and/or conditions, for example for athletes on special diets or following specific medical conditions [2]. A few ergogenic substances frequently found in DSs, such as creatine [3] and caffeine [4], also have the potential to provide small but significant performance effects. In general, the use of DSs is common in many sports, with a reported prevalence between 10 and 100% depending on the definition of DSs, the country, sport discipline, and competitive level [5,6]. A recent study examining supplement use among Norwegian athletes by using information reported by athletes on doping control forms found that 51% of the doping control forms contained information about athletes’ use of at least one DS during the seven-day period prior to the doping control [7]. 

In addition to the possible benefits of DSs, athletes and their support personnel should acknowledge that the use of these products is not without risks, as they may result in inadvertent negative health effects [8] and impaired recovery and performance following exercise [9,10]. Moreover, inadvertent doping from DSs containing substances prohibited for use by athletes according to the international anti-doping regulations continue to constitute a major problem [11,12]. Despite continuous efforts by anti-doping organizations, athletes still fail to check their supplements properly, resulting in anti-doping rule violations (ADRVs) [12,13]. To address the problem with doping substances in DSs, the World Anti-Doping Agency (WADA) recently established the Taskforce on Unintentional Doping to provide expert advice, recommendations, and guidance to prevent unintentional doping among athletes, including following supplement use [14]. It has also been suggested that regular supplement use may indirectly increase the risk of using prohibited substances through increasing the likelihood of developing liberal doping attitudes [15,16]

Overall, athletes in individual sports more often report using DSs than team sport athletes [7,17], while the latter seem more inclined to use products which could be described as high-risk in terms of containing both declared and undeclared prohibited substances [7]. In line with this, a Norwegian study found that positive doping tests linked to supplement use were predominantly present among team sport athletes [12]. 

Ice hockey is among the most popular team sports in Norway. The Norwegian Ice Hockey Federation is one of the fifty-five sport federations organized under the Norwegian Olympic and Paralympic Committee and Confederation of Sport and is thus subject to national and international anti-doping rules and regulations. In the period of 2003–2019, 5.2% of all doping samples collected by Anti-Doping Norway in Norwegian sport were from ice hockey players [18]. Moreover, ice hockey was among the Norwegian sport federations with both the highest absolute number of ADRVs and ADRV-to-sample ratios in the same period [18]. According to another recent study, ice hockey was the team sport with the highest reported prevalence of DS use among Norwegian athletes [7]. Even in the premier division, many ice hockey players in Norway combine their sporting careers as elite athletes with regular jobs to meet income needs. For such semi-professional team athletes, time management and non-athletic demands can lead to practical challenges that can contribute to less vigilance for anti-doping rules [19,20]. 

This study examines the culture of DSs from an anti-doping perspective through focus group interviews of male elite ice hockey players with the aim of obtaining a deeper knowledge about the attitudes, motivations, and practices relating to DS use, perceived doping risks, and athletes´ sources of inspiration and advice when considering using DSs.

## 2. Materials and Methods 

### 2.1. Research Approach and Ethical Consideration 

A qualitative interview study was conducted to explore the attitudes and practices surrounding DS use among elite athletes in ice hockey. Data were gathered from focus groups in order to gain in-depth insights into the research topic through group dynamics and collective input.

This study was approved by the Norwegian Center for Research Data (reference number 642391). Prior to participating in the focus group interviews, each participant signed a declaration of voluntary informed consent and was given the option to withdraw their contributions to the study until the analysis was completed. Participants were informed that their data would be anonymized and treated confidentially.

### 2.2. Participants and Recruitment

The inclusion criteria were male athletes competing in the Norwegian Elite Hockey League and who were between 18 and 50 years of age. A total of 25 male elite ice hockey players aged 20–36 participated in the study. The recruitment of athletes was conducted in collaboration and in agreement with the Norwegian Ice Hockey Federation. Contact was set up with the sport director, physiotherapist, or a medical care representative from selected clubs who further invited athletes to participate in the focus groups.

### 2.3. Data Collection

The interviews took place from November 2022 to January 2023 and were conducted at the home arena of each team to provide a comfortable and familiar environment for the participants. One focus group was set up per club. In total, five focus group interviews were conducted with 4–6 athletes per group. On average, each interview lasted for 57 min (range: 45–60 min).

A semi-structured interview guide was developed and evaluated in two pilot interviews before being used in all five focus group interviews (Table 1). A semi-structured interview format was chosen to avoid restricting the exchange of knowledge among participants and to be open to unexpected turns. The interview guide consisted of open questions about the athletes’ (a) knowledge, use, and attitudes towards DSs, (b) sources of information on the effects and risks of using supplements, and (c) thoughts about anti-doping work. In the focus groups, the participants were also asked about the use of pharmaceuticals. Data about the use of pharmaceuticals are published elsewhere [20].

The interview process was completed when the questions in the interview guide reached saturation [21], which was accomplished after five interviews. The same interviewer/researcher conducted the two pilot interviews and all of the five focus group interviews. In addition, a research assistant attended and assisted with all interviews.

No participants were removed from the collected data or withdrew during or after the interviews. In cases where one or two individuals in a focus group were much more active during the interview at the expense of their teammates, the interviewer sought to counteract this by engaging and asking questions directly to the more quiet or passive participants so that all participants became active during each focus group.

### 2.4. Data Analysis 

The interviews were recorded digitally and transcribed verbatim. Interviews were conducted in Norwegian. A structured, thematic analysis method was used to identify themes, patterns, and meaning from the transcript of each focus group interview (*n* = 5) [22]. A thematic analysis is a six-step method [22]. In the first step, the data were familiarized by repeatedly reading the transcription. In the second step, the text was coded to obtain relevant elements of information related to the research question. The analysis followed a data-driven approach, wherein codes were generated based on the content of the data. In the third step, the codes were organized into potential themes that describe the research question. In the fourth step, the themes were reviewed to see if they corresponded to the codes and the entire data set. In the fifth step, the themes were named and given a summary. The sixth and last step was to present the analytic findings as an analytic text. Examples of extracts from the transcript-formed codes, subthemes, and main themes are presented in Table 2. The results of the analysis with the main themes, subthemes, and codes are presented in Table 2.

All quotes presented in the article were translated from Norwegian to English by the authors. The ice hockey players (IHPs) providing the quotes are identified by participant number in the text. 

## 3. Results

### 3.1. Overall Use of Dietary Supplements

Open-ended questions were asked about which types of DSs the athletes consumed, the underlying reasons and intentions for use, and the athletes’ perceptions regarding the doping risks associated with consuming these supplements and of DS use in general (Table 2).

Altogether, vitamin and mineral supplements, also categorized as medical supplements [5] (vitamin C, vitamin D, multivitamins, magnesium, iron, zinc, electrolytes, and Omega-3), sports products (whey protein and protein powder, gainer, sport drinks, and energy drinks), ergogenic substances (beetroot powder, beta-alanine, creatine, and caffeine tablets), and mixed products (pre-workout) were mentioned during the interviews. The athletes´ perceptions of DS prevalence in their sport and within their own team varied from “everybody uses it” to two or three athletes on the team using DSs.

Factors influencing the athletes’ decisions to use DSs were encouragement from the team coach and availability of supplements, such as through sponsorship agreements. The athletes frequently emphasized practical reasons why they used a DS (for example, using a supplement was seen as a time-effective way to ingest nutrients). Dietary supplements were often used to maintain a stable body weight and to ensure sufficient lean mass to meet the physical demands of the sports, particularly in terms of speed and explosiveness, without becoming too heavy.

The athletes expressed high confidence in the safety of DSs related to the doping risk. Teammates, coaches, and other support personnel were often used as expert sources when the athletes considered using a DS and when evaluating their efficacy and risk of containing prohibited substances. Domestic brands and products were considered as safe in terms of negative health consequences and the risk that they could contain substances on the WADA Prohibited List, whereas they were more skeptical about DSs obtained abroad when traveling to other countries.

### 3.2. A Busy Schedule and Sponsorships with Supplement Companies Facilitated DS Use

The ice hockey players highlighted that ensuring adequate nutritional intake posed a common challenge in their everyday routines. As many players were semi-professional athletes, they had busy schedules in their day-to-day lives, all while performing at the highest national level in their sport. In this context, the use of DSs became a nutritional strategy to cope with high energy demands and inadequate available time to eat regular and well-composed meals. As two of the ice hockey players explained, “It becomes a way to ingest nutrients when you train a lot instead of having to resort to large meals” (IHP 14) and “For my part, I take a protein shake after training because I find it difficult to eat after a workout. That is easier than eating food, as you can consume it quickly” (IHP 4). Another athlete explained that he considered DSs as important for recovery after training and competitions (IHP 12).

Sponsor agreements with nutritional companies increased the availability of various supplements and could reduce the threshold to use DSs. Athlete 21 added that when the team was sponsored by a nutritional company, DSs were freely available to everyone at no cost, and thus, the threshold to use DSs was lower. However, when the sponsorship expired, the athletes had to cover the expenses themselves. As some were unwilling to spend their own money on DSs, in his experience, this resulted in a decreased use of DSs.

### 3.3. Using Dietary Supplements as a Nutritional Strategy to Maintain Body Weight and Increase Muscle Mass

The athletes repetitively explained the importance of consuming enough nutritional calories, and several participants expressed concerns that an inadequate energy intake could have a negative impact on performance. Athlete 18 explained, “I take multivitamins, omega 3 and vitamin D. And pre-workout… I must check whether that is legal. Proteins, creatine, hmm… zinc, magnesium—that is all. Nothing more than that […]. I noticed that last year, when I did not take them for a long time, I often had to sleep after training, and I was more prone to minor illnesses compared to now when I take all the supplements I mentioned before”.

A recurring theme during the interviews was that young players explained that they had been encouraged to increase their body weight and muscle mass to meet the physical demands of the sport. In contrast, their older teammates often received advice against further weight gain: “When you become an ice hockey player, as a youngster, the only thing you are always told is that you must put on weight and get stronger. You are always told this. The first step as a hockey player is the physical part. And it takes time. There are very few 18–19 years-olds who have the required physique. You are told that you must gain weight and get stronger… And then you resort to that sort of thing [DS] a lot more”. (IHP 21).

More players recognized the benefits of DSs in relation to muscle building, such as creatine and protein powder, and the coaches recommended these supplements to the athletes to increase their muscle mass. As two athletes explained, “If you are in a build-up period and aiming to gain 3–6 kg to be able to compete at the level you want […] So with proteins and creatine, you will be able to gain a little weight, but not everyone should to do that”. (IHP6) and “We were told that you could use it [creatine] in the summer when there is an emphasis on strength training and when you are supposed to build muscle mass”. (IHP 25).

The athletes also discussed the pressure of maintaining the correct body weight in relation to performance. One focus group explored how body weight was related to a player’s position. They explained that the ideal weight varies for each player depending on the playing position. For example, IHP7 stated that players frequently involved in physical duels and confrontations tend to weigh more than other players. However, for players in positions requiring speed and endurance, a lighter weight is beneficial. Another player shared that he felt pressure from the coach to achieve a specific weight. As a result, he consumed less food than he previously had while he played in less professional teams. Player 5 expressed the pressure to maintain an ideal body weight: “If you gain too much weight, you will hear it”. Player 10 talked about a period when he ate fewer carbohydrates because he wanted to lose weight, but after a while, he noticed that his energy levels were lower and his performance decreased. Similar experiences were shared by other athletes.

Despite the fact that the athletes often emphasized and shared the opinion that DSs should not replace regular food, their actions showed a different practice, as exemplified by IHP 12: “That’s where the supplements come in, and the protein powder. You should get enough protein. You eat less food, or an appropriate amount of food and then you supplement with a protein shake high in proteins, so that you get the proteins you need with very few calories” (IHP12).

### 3.4. Using DSs to Optimize Performance

The athletes explained how they used DSs for the optimization of performance. Two athletes talked about their intentions to use DSs: 

IHP 6: “You always try to optimize no matter where you are in your career”. 

IHP 3: “Yes, exactly!” 

IHP 6: “You think that now I am here, it is going really well, then you always think that there is something that can make me get better”. 

IHP 3: “Yes, it is such a continued quest to improve. The body must be as prepared as possible for what is to come. I think that is what matters the most”. 

IHP 6: “Then it is not like the body gets any better with age when you are an athlete, you want to lubricate it as much as possible”.

Ice hockey player 10 mentioned that he used a pre-workout supplement (PWO) before heavy lifting as part of a strength training routine. He explained how he experienced the use of PWO: “Beta-alanine, it is beta-alanine that makes you feel a bit warm inside and stuff like that. Like fired up. You get a little more pump. You feel that you get a little more pump”.

### 3.5. Doping Risk and Sources of Inspiration and Advice on DS Use

To examine the athletes´ thoughts about DS use in relation to the risk of unintentional doping through ingesting substances on the WADA Prohibited List, the athletes were provided with a scenario in which a teammate had started using a DS and recommended it to them. They were then presented with four different alternatives for how and where the athlete had obtained the DS and asked how they would react in the different scenarios. The alternatives were the following: (1) DSs purchased from a Spanish shop, (2) purchased from a Norwegian shop, (3) received from a person in the athlete´s pool of support personnel (e.g., doctor, coach, physiotherapist, etc.), and (4) received from an allocated goodie bag.

The athletes expressed skepticism about DSs purchased abroad due to the risk of contamination and cited cases where athletes had tested positive for prohibited substances because of contaminated DSs. In contrast, most athletes considered DSs bought or produced in Norway to be safe. An athlete put it succinctly: “I don’t worry much if it is produced or sold in Norway” (IHP 5). Another athlete shared a similar opinion regarding products bought in Norwegian stores: “It is foolish of them [Norwegian stores] if they sell something illegal without knowing it […] It cannot be available in the store if there are any prohibited substances in it. If so, someone has made a mistake. Also, it is usually labelled on the product that it has been tested” (IHP 15).

In another focus group, the players discussed the degree of doping risk when the DSs were bought in Norway:

IHP 21: “I believe it is a bit safer. At least [brand name] is something you think you can trust. I would have been a little more worried if someone had bought it at [Store name] from across the border. But, yes, I also think that it is safe. Although I have learned that you should never rely entirely on the content and safety of DS, because they may become contaminated. But you feel that it is safe”.

IHP 22: “I feel the same way. But that is partly because everyone claims that yes, it was bought in Norway, so it is probably fine”. During the discussions about how the athletes would approach DSs received in goodie bags or as prizes, some players indicated that they would likely first assess the type of product which was in the bag. One athlete admitted, however, that it would also be possible that he would forget to consider the risks and just consume the product without any further consideration. Maybe he would not even consider the product to be a DS.

When asked about the sources they used for nutritional advice, the athletes explained a lack of professional advice and guidance from support personnel with expertise in this area. One player explained that he used an app to track his caloric intake, while another sought advice from one of the team’s physiotherapists. 

If the athletes had received a DS from an athlete support personnel, they were confident that it had been thoroughly checked and “approved” by the support personnel, meaning that further investigations from the athlete´s side were unnecessary. An athlete expressed complete trust in his physician, saying, “If the doctor gave me something, I would have taken it” (IHP 1). Along the same lines, other athletes believed that their athlete support personnel would ensure the safety of the products before providing them to the athletes. 

The athletes also placed considerable trust in each other. Several said they would buy what others buy and that there is a mutual understanding within the team on what are considered safe supplements. Products that have not previously triggered a positive doping test were mostly labeled as safe by the athletes. As one athlete put it, “It is easier to take it if there are others on the team who take it, so, then there is a kind of common understanding that that supplement is okay” (IHP 17).

The players also talked about how cultures could vary between countries, and IHP 6 problematized that they trusted what fellow athletes and role models did:

IHP 6: “Let say a top athlete from the NHL [National Hockey League, US] uses a product”.

IHP 3: “[Mentioned a player]”

IHP 6: “Yes, for example. How many would then have bought the product?”

IHP 1: “Yes!”

Some players mentioned players in the NHL (National Hockey League, the largest ice hockey league in North America) being ambassadors and acting as influencers for different DS brands and products and argued that if the products work for some of the best players in the world, the products probably work for them as well.

Although several of the participants knew that as athletes, they have full responsibility for what they put in their own bodies according to the anti-doping rules, they still expressed a dependence on support staff for checking DSs. This was mainly because they themselves did not know where to find the information they needed to carry this out or how they should examine a supplement´s possible risks in practice. In one focus group, they explained that all players on the team were required to complete a basic anti-doping e-learning program provided by Anti-Doping Norway once a year. However, they also admitted that they mostly completed it because it was mandatory, and that they were not that interested or aware in the actual content of the program. 

When asked where they would check whether a DS was safe to use, many players clearly demonstrated a level of uncertainty and a general lack of knowledge. Through their discussions, they revealed that they themselves had probably not made use of the various resources made available for athletes to avoid unintentional doping, although they gave general references to the fact that such tools probably existed. In one focus group, the athletes suggested using various anti-doping resources:

IHP 1: “I would perhaps use the anti-doping web page”.

IHP 2: “Yes, WADA or anti-doping [Anti-Doping Norway] probably have something”.

IHP 5: “Maybe looked at the back of the packaging or the label about what it contains. On the product”.

IHP 1: “Surely there must be something like that”.

IHP 2: “Is there a list?”

IHP 3: “Like a do not use label?”

IHP 1: “Yes, maybe. On protein powders there is a label which says whether the product is approved by Anti-Doping Norway, or? I think it is like that.” 

When the players discussed whether the use of dietary supplements could be a gateway to more potent, prohibited substances, there were different opinions. Some argued that if one experiences an effect from protein powder and creatine, it could make them want to try other products. However, they did not think this issue was relevant to top-level athletes, who should know about the risks behind the use of such products. Instead, they said it could be a problem, for example, among young individuals who mainly exercise at a fitness center. 

## 4. Discussion 

In this study, male ice hockey players competing in the elite division in Norway participated in focus group interviews to provide insights into the culture surrounding the use of DSs in this sport. 

Athletes use DSs for several reasons, such as improving athletic performance, improving health, and accelerating recovery [5,6,23]. While females tend to focus on the health benefits of supplements, males more often use DSs to enhance performance [6]. In line with this, our findings suggest that male ice hockey players used DSs as a quick and practical way to increase or maintain body weight in addition to increasing lean body mass, especially during off-season periods with heavy strength training. Many struggled to follow the general recommendation for optimal nutrition and consequently turned to DSs as a strategic approach to meet their professional ambitions in the sport. Encouragement from athlete support personnel, in particular the coach, and the increased availability of supplements through sponsor agreements were identified as key facilitators for supplement use.

A common theme among the athletes was pressure to achieve an “ideal weight” for optimal performance in the sport. This aligns with earlier studies that emphasize the importance of fitness characteristics for all positions in ice hockey [24]. While the athletes in this study emphasized food as the primary source of nutrients, they considered dietary supplements as helpful and, in some cases, necessary aids in achieving weight-related goals and maintaining energy levels. The importance of protein supplements and creatine related to weight gain and building muscle mass was highlighted by most athletes. 

The use of DSs among athletes is widespread in most sports [5,6] even though most experts agree that optimal nutrition plays a far more significant role in performance [2]. The appropriate use of DSs could offer small but significant benefits to some athletes, but also introduces the possibility of adverse consequences [25]. This study focuses on the use of DSs in an anti-doping context from the perspective of an anti-doping organization. It is well documented that DSs may contain substances which are prohibited to be used by athletes according to the anti-doping policies in sports [26], and the use of such products may lead to an athlete submitting a positive urine or blood sample, resulting in an ADRV [12,13]. Prohibited substances have been found in all types of supplements, e.g., [27], but the risk of doping seems particularly high when using supplements claiming to have performance-enhancing effects [26,28], such as multiple-ingredient PWO supplements [12,29]. The athletes in this study reported frequent use of various types of supplements, of which most were medical supplements, sport products, and ergogenic substances, which is in line with quantitative studies on supplement use among Norwegian ice hockey players [7] and other research on athletes´ supplement use [6]. There is no universal guide which presents the doping risk per DS category; however, recent research suggests that medical supplements, sport products, and single-content ergogenic supplements (e.g., caffeine) could be considered to have a low to medium doping risk [12,26,28,30,31]. However, high-risk supplements, such as PWO, were also used, particularly in relation to strength training sessions. 

The players shared stories of banned athletes and expressed a fear of ingesting a contaminated supplement. Although the athletes were aware of the possible risk that DSs may contain prohibited substances, they demonstrated a relaxed attitude towards this when it came to their own use. Through their actions, the athletes showed little degree of vigilance in relation to the possible risk associated with supplement use. Most did not properly check the safety of the supplements they consumed, which is similar to what has been reported in other athlete cohorts [32]. The main reason for why the athletes did not check their supplements in terms of their safety and doping risk was because they argued they did not know how and where to check. 

Most athletes displayed a fundamental trust in the advice and recommendations provided to them from the coach and other support personnel and relied on them to oversee and manage their supplement intake. Athletes in general expressed a lack of access to qualified advice from personnel with expertise within the field of sport nutrition. Even though they competed at the highest national level in ice hockey in a physically demanding sport, none of the athletes had previously received personalized dietary counseling. Instead, the coach was identified as an important influence on supplement practices and a key source of information regarding DS use, which is in line with previous studies [33,34,35]. The dietary advice the players received from the coach seemed basic and general, such as when the coach promoted the use of creatine to enhance muscle growth and improve physical strength.

Supplements acquired domestically were considered safe in contrast to foreign products, of which many of the athletes were skeptical, particularly with regard to the risk of them containing prohibited substances. In addition to the coach, the athletes relied on each other to find out which products were safe to use, and they relied on others around them to have conducted the necessary research. This is in stark contrast to the WADA’s anti-doping rules, which emphasize the principle of strict liability and clearly state that athletes are fully responsible for any prohibited substance in their bodies [36].

When asked about the preventive anti-doping education the players were required to complete by their club, it did not seem like they assigned much importance to this. Although some studies have reported somewhat promising results on athletes´ knowledge, attitudes, and behavior following anti-doping e-learning [37], the long-term effects are uncertain. This underscores the importance of continuing to carefully assess both the immediate and long-term effects of specific anti-doping education and prevention efforts [38].

## 5. Limitations

Using focus groups as a method has some inherent limitations. The selection of information in group discussions can give new insights but can also limit what and how the participants discuss. The participants can unwillingly agree with what others in the group state without meaning it. This might have occurred in the current study, as participants frequently answered “yes” or “agree” to what others said, serving as a support for the statement.

The qualitative approach provides a deeper insight into the topic than what would be possible using quantitative research. On the negative side, the findings may not be representative of the population from which the sample was taken. The data presented in this study are based on interviews with five Norwegian ice hockey teams out of ten teams total in the elite national hockey league. However, the fact that data saturation was met after five interviews, and that the athletes´ experiences and attitudes agreed well across the interviews, suggests that the findings presented here are applicable to elite ice hockey players in Norway in general.

## 6. Conclusions

In summary, the present study highlights the complex interplay of factors influencing dietary supplement use among athletes. The primary motivations for using DSs among the interviewed elite-level ice hockey players were to optimize performance, regulate weight, and ensure adequate nutrition. Although they were aware of the risks associated with DS use and their own responsibility to mitigate these risks in line with the anti-doping rules, the players expressed a significant and uncritical level of trust in their support staff in general, and in the coach and physician in particular, when it came to controlling and recommending dietary supplements. This trust increases the risk of unintended doping, which can threaten the athletes’ careers and lead to exclusion from the sport.

Studies have revealed that a substantial portion of athletes rarely consult an expert in nutrition before consuming DSs [39], overestimate their perceived knowledge about DSs [40], and are not familiar with the anti-doping rules in relation to the risks of supplement use [33]. This study underpins the importance of further raising awareness and knowledge among both athletes and support staff to secure the use of dietary supplements. The findings suggest that athletes taking a mandatory anti-doping e-learning course is not sufficient to promote safe and informed decisions regarding supplement use. When considering using DSs, athletes should be advised to carefully assess the need, effects, and risks associated with DSs, preferentially under the guidance of qualified personnel [25]. As there are a myriad of DSs on the market with different contents and claimed effects, each DS should be examined individually.

## Figures and Tables

**Table 1:** Interview guide.

Dietary supplements - What do you think of when we speak about dietary supplements? - Can you talk about the eventual use of dietary supplements among the athletes on your team? ⚬ Do you feel that dietary supplements are a necessity and something athletes “must take”? ⚬ Which dietary supplements do you think are most used in your sport? ⚬ If you feel it is comfortable to talk about it, can you talk about your own use? - Where do you get knowledge and information about dietary supplements? ⚬ Can you remember specific situations where you sought information. ⚬ Scenario: A teammate has bought a new dietary supplement and shares positive experiences with the effect of it. You want to try it and ask where he bought this. We will now discuss the various answer options he can give: Bought in a Spanish store Bought in a Norwegian store Got it from Athlete Support Personnel Got a goodie bag Anti-doping - What do you think about claims that dietary supplement can be a gateway to doping? - How do you experience other athletes (role models) use of supplements? ⚬ What do you think is your responsibility ⚬ What do you think the coaches and support teams responsibilities are?

**Table 2:** Overview of inductive descriptive thematic analysis. Interview extracted produce codes, which were then grouped into subthemes.

(a) Examples of how interview extracts produce codes, which are then grouped into subthemes.		
Interview Extract		Codes (subtheme)
“Trusting Norway a bit blindly. Maybe a little naive, but that is how I operate anyway. I do not take that much, but the creatine that I use is what I think about. Know several people who use that type in Norway, and no problems for them, so I see it as okay then”.		Norwegian products are safe (doping risk). Trust each other when choosing which supplements to use (doping risk). Ergogenic products (dietary supplements being used).
“Beta-alanine, it is beta-alanine that makes you feel a bit warm inside and stuff like that. Like fired up. You get a little more pump. You feel that you get a little more pump”.		Optimize performance (intentions with the use of dietary supplements). Ergogenic products (dietary supplements being used).
(b) Subthemes and codes produced for use of dietary supplements among male ice hockey players.		
Main theme	Subtheme	Codes
Use of dietary supplements	Dietary supplements being used	- Vitamins and minerals - Sports products - Ergogenic products
	Facilitators for the use of dietary supplements	- Influence by the coach - Individual reasons - Time and availability - Age - Culture
	Intentions for the use of dietary supplements	- Optimize performance - Stay healthy - Nutritional strategies - Maintain the right body weight
	Doping risk from using supplements	- Norwegian products are safe - Trust in each other - Trust in athlete support personnel

## Data Availability

The anonymized dataset is available on request from the authors.
